# Understanding developmental system drift

**DOI:** 10.1242/dev.203054

**Published:** 2024-10-17

**Authors:** Áine McColgan, James DiFrisco

**Affiliations:** ^1^ Theoretical Biology Lab, The Francis Crick Institute, London NW1 1AT, UK; ^2^ Department of Life Sciences, Imperial College London, London SW7 2AZ, UK

**Keywords:** Developmental system drift, Robustness, Compensatory evolution, Evo-devo, Gene regulatory networks, Model organisms

## Abstract

Developmental system drift (DSD) occurs when the genetic basis for homologous traits diverges over time despite conservation of the phenotype. In this Review, we examine the key ideas, evidence and open problems arising from studies of DSD. Recent work suggests that DSD may be pervasive, having been detected across a range of different organisms and developmental processes. Although developmental research remains heavily reliant on model organisms, extrapolation of findings to non-model organisms can be error-prone if the lineages have undergone DSD. We suggest how existing data and modelling approaches may be used to detect DSD and estimate its frequency. More direct study of DSD, we propose, can inform null hypotheses for how much genetic divergence to expect on the basis of phylogenetic distance, while also contributing to principles of gene regulatory evolution.

## Teaser

**Summary:** This Review examines how developmental system drift, or divergence in the genetic underpinnings of conserved traits, can be studied to inform comparative developmental biology.

‘*The genetic link between homologous structures cannot be analysed down to individual genes, but must be based on the gene-complex or such portions of it, or groups of genes, which control the structure in question. The individual members of these groups of genes may, during phylogeny, become changed by substitution, addition, or loss, so that…the groups may come to contain few or none of their original members.*’

Sir Gavin De Beer (1938), p. 38

## Introduction

In developmental biology, we often rely on a limited set of well-characterised model organisms, operating under the assumption that conserved phenotypes – or homologues – imply conserved genetic architectures. Highly conserved genetic mechanisms have been identified for several cell types and developmental processes (Davidson and Erwin, 2006; Peter and Davidson, 2011; Prud'homme et al., 2007; Wagner, 2007, 2014; Arendt et al., 2016; DiFrisco et al., 2020; Hobert, 2021). However, there is also mounting evidence that conserved traits can diverge in their genetic underpinnings over evolutionary time, a process known as developmental system drift (DSD; see Glossary, Box 1) (True and Haag, 2001; Weiss and Fullerton, 2000; Haag and True, 2021). When DSD has occurred, the genetic mechanism for one trait is not shared for homologous traits, and assuming as much leads to error. While the exact frequency of DSD is unknown, individual studies have found it in a wide diversity of organisms and biological processes, including the vertebrate segmentation clock (Krol et al., 2011), nematode vulva development (Hoyos et al., 2011) and insect gap gene networks (Wotton et al., 2015). Understanding how DSD occurs is crucially important for the practice of generalising from model to non-model organisms, and forms part of a broader effort to establish patterns of conservation and variability for conserved developmental traits and underlying mechanisms (Moczek et al., 2015).

**Box 1. Glossary**

**Developmental system drift (DSD).** Divergence in the genetic basis of conserved traits over evolutionary time (also known as system drift and phenogenetic drift).

**Directional selection.** Selection in which the trait optimum is an extreme value, which tends to move the population trait mean in the direction of that extreme.

**Evolvability.** The capacity to generate potentially adaptive variations.

**Gene regulatory network (GRN).** A set of genes in which at least some genes regulate the expression of other genes by transcription factor proteins binding to *cis*-regulatory elements.

**Genetic drift.** Random fluctuation in allele frequencies in a population due to sampling finite numbers of gametes from one generation to the next.

**Qualitative DSD.** Developmental system drift involving change in the identity of genes.

**Quantitative DSD.** Developmental system drift involving a change in gene expression levels or regulatory dynamics without a change in the identity of genes.

**Robustness.** The stability of a phenotypic attribute to genetic perturbation (genetic robustness) or environmental perturbations (environmental robustness).

**Stabilising selection.** Selection in which the trait optimum is a particular intermediate (non-extreme) value, which tends to ‘stabilise’ the population trait mean at that value.

Although DSD presents a complication to comparative work, it also presents new opportunities. It encourages increased taxonomic diversity in model organism research, which would improve the quality of comparative developmental biology. We are currently in an unprecedented age of data availability, yet research in development often remains pragmatically bound to experimentally tractable model organisms. Demonstrating – rather than assuming – patterns of conservation or variability in genetic mechanisms requires comparisons of large amounts of data across multiple species to detect with confidence. These comparisons can shed light on the subtle ways species differ at the genetic level, in ways that commonly used methods such as gene ontology (GO) annotation would not detect. Developmental data from a broad range of organisms can then be used to inform null hypotheses for the amount of evolutionary change we expect for different traits (Church and Extavour, 2020). We suggest that consideration of DSD is key to building such hypotheses, because it alters the extent to which we can rely on evidence of character homology to predict conservation of underlying gene regulatory networks. This could also have a translational impact in the biomedical sciences, including in drug trial research (Lynch, 2009).

The key to incorporating DSD into null hypotheses for evolutionary change is to combine this more diverse taxonomic sampling with theoretical understanding of how DSD occurs. We find that there are two primary mechanisms for how a trait can remain stable while its genetic underpinnings change. One results from the robustness (see Glossary, Box 1) of developmental gene regulatory networks to mutations in some of their components (de Visser et al., 2003; Wagner, 2005; Payne and Wagner, 2015). Robust networks inherited from a common ancestor allow genetic changes to accumulate in the descendant lineages. Another mechanism for DSD is compensatory evolution by natural selection (Haag, 2007; Pavlicev and Wagner, 2012; Rajon and Masel, 2013). When two developmental processes in the same organism are pleiotropically correlated, adaptive change in one process can disrupt the other, necessitating compensatory changes to restore the disrupted process. Compensation can lead to complex and convoluted regulatory networks underlying conserved phenotypic outputs, as we discuss below.

This Review presents the major ideas, evidence and open problems arising from studies of DSD. We begin by clarifying what DSD is and how it may happen, and proceed to detail the many experimental findings of DSD. We propose ways to detect DSD using observational, perturbational and computational approaches, which will be essential to estimating its frequency. We then consider the broader implications of DSD for comparative evolutionary-developmental biology (evo-devo).

## What is developmental system drift?

The term ‘developmental system drift’ was originally coined by True and Haag (2001), who defined it as ‘the process by which conserved traits diverge in their developmental genetic underpinnings over evolutionary time’. This definition can be refined with further distinctions, with the aim of using terms to track different mechanisms of DSD and helping to transform DSD from a label for a phenomenon into a process that can be studied. The central aspects meriting further clarification include: how DSD differs from related phenomena, such as drift and robustness; what exactly the ‘conserved traits’ are; which kinds of divergence or genetic change should be considered DSD; and whether DSD is evolutionarily neutral or adaptive.

### Developmental system drift is distinct from drift and robustness

Developmental system drift is distinct from genetic drift (see Glossary, Box 1), which is defined as the random fluctuation in allele frequencies in a population. Drift is a necessary consequence of sampling finite numbers of gametes from one generation to the next (Walsh and Lynch, 2018, p. 25). It is one of the five major forces of population genetics alongside selection, mutation, recombination and migration or gene flow. In contrast to DSD, genetic drift is defined at the genetic level rather than describing a genotype-phenotype relationship, and is not necessarily associated with a conserved phenotype. Although the term ‘drift’ is included in ‘DSD’, it is important to recognise that drift is just one possible population-genetic mechanism that can contribute to DSD, the others being mutation and selection.

DSD is also distinct from genetic robustness, defined as the stability of a phenotype to perturbations of its genetic causes (de Visser et al., 2003; Payne and Wagner, 2015). A population, individual or trait can be robust in this sense. By contrast, DSD involves the comparison of genotype-phenotype relationships across multiple populations, species or higher-level taxa. Robustness is a system property, whereas DSD is an evolutionary outcome. Robustness can contribute to DSD, but robust systems do not automatically undergo DSD. DSD can also occur by selection on pleiotropically correlated traits followed by genetic compensation, a process that requires that the traits are not genetically robust (see the section ‘DSD can be neutral or adaptive’).

### DSD requires trait homology

DSD happens to ‘conserved’ traits rather than to unrelated traits or convergently evolved traits (homoplasies). It is not surprising that convergent phenotypes should have different genetic underpinnings if they evolved from independent starting points. What makes DSD interesting is that it violates the expectation [historically common in evolutionary developmental biology (evo-devo) and comparative developmental genetics (Church and Extavour, 2020; Moczek et al., 2015; Davidson and Erwin, 2006)] that conserved traits should also have conserved genetic mechanisms. But what exactly does it mean for a trait to be conserved? Which kinds of change can a trait undergo (e.g. size, shape, heterochrony, etc.) while still being conserved, or ‘the same’?

Fortunately, these issues are largely addressed by a rich conceptual framework surrounding the concept of homology, which combines insights from morphology, systematics, embryology and developmental genetics (Hall, 1994; Wagner, 2007, 2014; DiFrisco et al., 2020; DiFrisco, 2023). Work on homology has identified criteria that are indicative of shared ancestry, including sameness of position in the body plan, and complex similarities in the phenotype and in genetic mechanisms that are unlikely to be independently evolved. Homologies are invariant to changes in size, shape and heterochrony, as well as in function. ‘The tetrapod limb’ is viewed in this light as a conserved body part, despite the many forms and functions it can take. We suggest that studies of DSD should rely on homology for interpreting and implementing the ‘conserved trait’ part of the definition.

It is also important to consider the organisational level or scale at which a phenotype is defined when assessing homology and DSD, because a developmental process can be conserved at one level but not at another (de Beer, 1938, 1971; Riedl, 1978; Raff, 1996; DiFrisco, 2023). For example, in insect segmentation, many of the morphological segmental identities along the anterior-posterior (AP) axis are conserved, while the processes involved in generating them (i.e. sequential versus simultaneous segmentation) are not (Clark et al., 2019). Genetic divergence despite the conservation of segmental identities is DSD, but this does not imply DSD in the segmentation process. Here, again, existing thinking about homology at different levels of organisation can be a helpful guide (DiFrisco and Jaeger, 2021; DiFrisco, 2023).

### Which kinds of genetic change constitute DSD?

DSD is a divergence in the ‘genetic underpinnings’ of a trait. It is, therefore, restricted to genetic changes rather than other kinds of developmental changes. For example, the embryological precursors of a structure can diverge despite the structure being homologous – such as the different gastrulation modes that lead to a gastrula, a fact that has long been appreciated in comparative embryology (Spemann, 1915; De Beer, 1938, 1971). These developmental changes are indicative of DSD in underlying genes, but are not themselves DSD because they are not genetic changes.

**Fig. 1.**
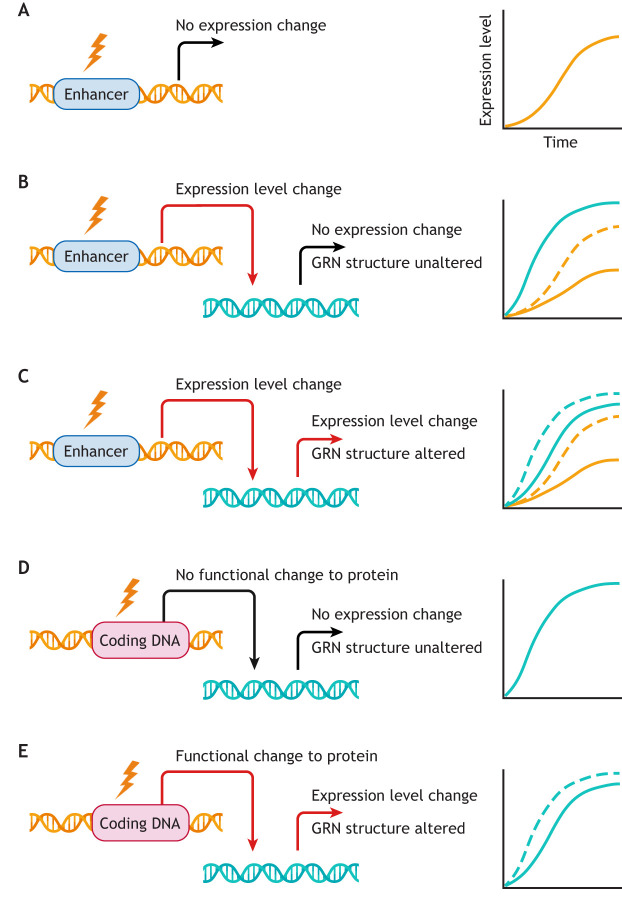
**Developmental system drift can occur at different points in a gene regulatory network for a given trait.** Each case of developmental system drift (DSD) involves change(s) at one or more levels of control (e.g. enhancer sequence, transcription-factor binding) despite the stability, or lack of change, at another level [e.g. gene expression or the trait controlled by the gene regulatory network (GRN)]. (A) Changes in a *cis*-regulatory element (CRE) that do not change the gene's expression level (solid orange line). (B) Changes in a CRE that change the gene's expression level from the original state (dashed orange line) but not its effect on expression of other genes in the GRN (solid teal line). (C) Changes in a CRE that change the gene's expression level from the original state (dashed orange line) and its effect on other genes in the GRN (dashed teal line), but not the trait controlled by the GRN. (D) Changes in a protein-coding region that do not change the gene's binding efficacy or the expression level of other genes (solid teal line). (E) Changes in a protein-coding region that change the expression level of other genes (dashed teal line) but not the trait controlled by the GRN. Created in BioRender. McColgan, A. (2024) BioRender.com/q95r284.

A gene can change biochemically in *cis* or in *trans*. This allows distinguishing DSD at different levels of a gene regulatory network (GRN; see Glossary, Box 1) (Fig. 1). There are two primary explanations for how a conserved phenotypic feature at a given level of organisation can be stable to genetic change. One is genetic robustness. Within the process of transcription, each regulatory step possesses some mutational robustness relative to other steps (reviewed by Aguilar-Rodríguez and Payne, 2021). Another explanation is stabilising selection (see Glossary, Box 1). In this case, genetic changes lead to aberrant phenotypes that are selected against because they deviate from some intermediate optimal value. Although stabilising selection on a trait or GRN output can fix the underlying regulatory mechanism, transcriptional networks will often possess extra degrees of freedom relative to the trait under selection, allowing them to find multiple ways to reach the same output. Deleterious change in one regulatory step can be compensated by change in another. Some studies have found extensive *cis-trans* compensation in mouse (Goncalves et al., 2012) and drosophilids (Landry et al., 2005), in line with models suggesting that gene expression is frequently under stabilising selection (Hodgins-Davis et al., 2015; Signor and Nuzhdin, 2018).

Assuming the function of a GRN is either robust or being maintained by selection, it is more likely for changes in *cis* regulation (Fig. 1A,B) to not change the phenotype compared to changes in protein-coding regions (Fig. 1D,E). Current evidence indicates that *cis-*regulatory elements (CREs) often have high redundancy and compensatory capacity, even for key developmental genes (Kim and Wysocka, 2023; Hill et al., 2021; Coolon et al., 2014; Hong et al., 2008), with many enhancers exhibiting greater cross-species variability than the associated promoters and coding regions (Villar et al., 2015; Andrews et al., 2023). Enhancers regulate transcription in a tissue- and time-specific way, depending on the presence of transcription factors and the state of chromatin accessibility. For this reason, mutations in enhancers are thought to be less pleiotropic and thus less likely to be deleterious than changes in coding regions, which manifest everywhere the gene is expressed (Carroll, 2005; Paaby and Rockman, 2013). However, this is an active area of research, with evidence suggesting that many enhancers may also possess some degree of pleiotropy (Lynch and Wagner, 2008; Sabarís et al., 2019) and can be divided into modular versus pleiotropic CREs (Murugesan and Monteiro, 2023). A general implication is nonetheless that enhancers are more ‘evolvable’ than coding regions, providing a base of variability that can fuel adaptive evolution (Wray et al., 2003; Hill et al., 2021). A less well-appreciated point is that evidence for high enhancer evolvability is also indicative of high enhancer DSD.

Experimental reports of DSD more often focus on changes in transcription factors (TFs) and regulatory circuits (see below). This may be partly because TFs are much easier to identify than CREs using current methods (Halfon, 2017), or because changes in TFs and regulatory circuits without a change of phenotype are considered more surprising or significant. We see no reason to exclude changes in CREs as potential cases of DSD, even if such changes are expected to be pervasive. Increasing attention to CREs will also synergise with broader shifts in the study of GRNs from gene presence/absence to quantitative dynamics of gene expression (Briscoe, 2019; DiFrisco and Jaeger, 2019; Rand et al., 2021).

The dynamical behaviour of a GRN and its phenotypic output are additional points of robustness or stability that can allow underlying DSD to occur. Quantitative theoretical modelling has led to the finding that multiple network topologies are capable of producing the same dynamics (e.g. oscillation, bistable switching, etc.) (Jiménez et al., 2015, 2017). Moreover, distinct gene expression dynamics can give rise to the same phenotypic output, such as a stripe of gene expression (Cotterell and Sharpe, 2010). When selection acts on only the phenotypic output, this should allow genetic systems to mutate and drift along unconstrained degrees of freedom. This idea can be expressed in the language of dynamical models. Supposing that quantities of gene expression define the parameters of a multidimensional space, for any nonlinear system, there will be regions of that space where small expression changes have a large impact on system behaviour, and regions of stability where expression changes have little to no effect (Ingalls, 2013; Steinacher et al., 2016). The latter regions of stability define the range of parameter values where DSD can occur in the underlying transcriptional components.

Following Wotton et al. (2015), DSD involving changes in gene expression or regulatory dynamics without change in the identity of genes can be called quantitative DSD (see Glossary, Box 1), which has been found in the gap gene system of various dipterans (Wotton et al., 2015). Qualitative DSD (see Glossary, Box 1), or simply DSD, would then require change in the identity of genes. Most mentions of ‘DSD’ in the literature refer to qualitative DSD in this sense. In practice, most cases of DSD can be expected to involve a combination of both quantitative and qualitative changes.

### DSD can be neutral or adaptive

DSD can occur by neutral evolution or by selection. In neutral DSD, diverged species accumulate molecular differences that have no measurable effect on the phenotype or on fitness. The molecular variants ultimately arise from mutations, including insertions, deletions and gene duplications, and they spread in the population by drift. At the phenotypic level, most non-molecular traits are thought to be subject to stabilising selection (Schmalhausen, 1949; Lande, 1976; Kingsolver et al., 2001; Johnson and Barton, 2005; Hodgins-Davis et al., 2015). Under these conditions, DSD can occur when variations in the genetic underpinnings of a trait do not displace it from the optimum. This is a form of mutational robustness that can be amplified by redundancy in genomic architectures (see Haag, 2007; Lynch, 2007; DiFrisco, 2023). If an ancestral trait is regulated by two redundant mechanisms, for example, one or the other mechanism can be differentially lost in descendant lineages without deleterious fitness consequences and without change in the trait. However, neutral or ‘nearly-neutral’ DSD can also occur when new genetic variants are mildly deleterious but arise in populations where stabilising selection is not strong enough to remove them (Lynch, 2007; Lynch and Hagner, 2015). This process can be considered ‘neutral’, in that it is not driven by selection. Because selection is weaker in small populations, a reasonable prediction is that the rate of neutral DSD should be negatively correlated to population size and the strength of selection. The extent of neutral DSD should also positively depend on factors such as mutation rate and the phylogenetic distance (branch length) between compared groups (DiFrisco, 2023; see Lynch et al., 2011).

**Fig. 2.**
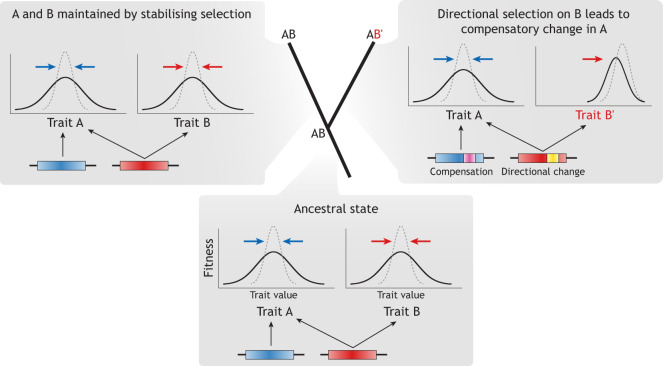
**Developmental system drift due to selection on pleiotropically correlated traits followed by compensation.** In the ancestral state (AB, bottom), traits A and B are under stabilising selection and are pleiotropically linked, such that trait A is influenced by change in trait B. In one descendant lineage (top left), there is no change of selection pressure, and A and B do not change. By contrast, in another descendant lineage (top right), trait B undergoes directional selection and changes to B′. Because the genetic basis of B is pleiotropically linked to A, directional change in B interferes with A. Stabilising selection on trait A then favours compensatory genetic changes in its genetic basis, allowing the initial trait value distribution of A to be maintained. As a result of this process, trait A remains conserved across both lineages despite having acquired different genetic bases – an example of developmental system drift (see Johnson and Porter, 2007; Pavlicev and Wagner, 2012; DiFrisco, 2023). In the trait distribution graphs, solid lines denote the actual population distribution and dotted lines denote the distribution favoured by selection, with arrows indicating selection pressures.

The earliest studies of DSD attributed it explicitly or implicitly to neutral evolution, as evidenced by the inclusion of ‘drift’ in the term (True and Haag, 2001; Weiss and Fullerton, 2000; compare with Schmalhausen, 1949). Given that DSD involves genetic variants that do not change the phenotype, they do not influence fitness either, and so selection should not be involved. This reasoning is valid for the conserved trait in focus whose genetic underpinnings change. But many genes are pleiotropic, affecting multiple traits or other genes (Zhang, 2023). If the same pleiotropic genes influence a trait subject to directional selection (see Glossary, Box 1) as well as a trait under stabilising selection, those genes can undergo directional change as compensatory genetic changes maintain the stabilised trait (Fig. 2). If we then compare a species that has undergone this process with a related species that has not, e.g. due to different selection pressures, the species can share conserved traits with different genetic underpinnings (Johnson and Porter, 2007; Pavlicev and Wagner, 2012; Haag and True, 2021). Interestingly, unlike neutral DSD, adaptive DSD requires that the trait undergoing DSD – the one subject to stabilising selection – is not robust to genetic change. The compensatory genetic changes must affect the phenotype in order to be selected (Pavlicev et al., 2011).

If most traits are subject to stabilising selection, then whenever directional selection is occurring and pleiotropic genes are involved, there is likely to be the above combination of stabilising and directional selection on different traits (Baatz and Wagner, 1997). Modelling and simulation studies have suggested that this kind of adaptive DSD can be rapid compared with neutral DSD (Johnson and Porter, 2007; Schiffman and Ralph, 2022). In practice, however, it is often unknown whether DSD that has been detected experimentally is neutral or adaptive, or some combination of the two. However, neutrality versus adaptation are distinguishable by different genomic signatures. We suggest that existing tests from the field of molecular evolution can be used here for disentangling neutral versus adaptive genetic change (see ‘Detecting and modelling DSD’ below). However, more-complete verification requires validation of the causal link between genes and phenotype.

## Experimental examples of DSD in metazoans

Although DSD may have been understudied to date, examples have nonetheless been identified across multiple kingdoms of life in a wide range of biological characters. Here, we examine a few key studies representing a diverse set of organisms, with the aim not to provide an exhaustive summary of all research to date but rather to highlight the differences in how DSD can occur and be detected. We focus on metazoans below, but note that DSD has also been found in plants (Gaudinier and Blackman, 2020) and in unicellular eukaryotes such as yeast (Tsong et al., 2006; Shapiro et al., 2012), and may even be implicated in the evolution of RNA interference (RNAi) in eukaryotes (Torri et al., 2022).

### Insects

While *Drosophila* is evolutionarily derived in comparison with other insect groups (Jaeger, 2011), they are extensively characterised and well studied, which makes them a useful model for the identification of DSD. An example is the *Drosophila* gap gene network, which is required for early embryo patterning and segmentation (Wotton et al., 2015). Despite being unusual for the majority of insect species, the form of long-germband development, where gap domains and pair-rule stripes are formed before gastrulation, appears to be largely conserved across the more recently diverged flies or Brachycera (Jaeger, 2011).

Examining the dipteran gap gene network using a combination of mRNA expression pattern staining and RNAi (RNA interference) knockdown of candidate genes, Wotton and colleagues found that early gap gene expression patterns differed between *Drosophila melanogaster* and the scuttle fly *Megaselia abdita*, with conserved positioning and timing of expression domains but notable differences in gene expression dynamics (Wotton et al., 2015; see Crombach et al., 2016). Despite this, both species end up with very similar gap gene expression patterns by the onset of gastrulation, suggesting that the overall patterning phenotype has been conserved and the genetic changes observed are a case of quantitative DSD, which compensates for changes in maternal inputs (qualitative DSD).

Another apparent case of DSD in early *Drosophila* development is in the Toll signalling pathway (Roth, 2023). The primary role of Toll signalling in the majority of metazoan lineages is in innate immune function, whereas bone morphogenetic protein (BMP) signalling is used to establish the dorsoventral (DV) axis in embryonic development. Roth (2023) has shown that while the immune function of Toll appears to be conserved across the insects (and other metazoan phyla), some insects, such as *Drosophila* and *Tribolium*, appear to have a reduced role of BMP signalling and an increased role for Toll signalling in DV axis formation (Pechmann et al., 2021). In particular, the Toll signalling pathway is essential for early BMP gradient formation in *Tribolium* (Nunes da Fonseca et al., 2010).

In *Heliconius* butterflies, Van Belleghem and colleagues compared the genetic architecture of wing colour patterning in two species that exhibit Müllerian mimicry. (Müllerian mimicry occurs when two or more species with effective evolutionary defences against a shared predator mimic each other's colour patternings or warning signals, to their mutual benefit.) Through profiling chromatin accessibility and gene expression, Van Belleghem and colleagues showed that the role of *optix* in red colouration was conserved in both species, yet the regulation of *optix* had diverged significantly (Van Belleghem et al., 2023).

This example also highlights the importance of linking DSD to a clearly defined trait (see above). In this case, genetic divergence has occurred in the regulation of the shared toolkit of wing patterning genes (particularly *optix*), and thus these patterning genes are the homologous trait that has undergone DSD (Van Belleghem et al., 2023). The colour patterns in the compared species are non-homologous, however, having evolved convergently; thus, genetic divergence underlying these traits is not an example of DSD.

### Vertebrates

DSD has been detected in vertebrates, most notably in the segmentation clock (Krol et al., 2011). Vertebrates as a clade are defined by possession of the vertebral column, which develops out of segmented precursors in the embryo known as somites. Somite boundaries are established by a molecular ‘segmentation clock’ consisting in the oscillating transcription of cyclic genes, interacting with a moving morphogen gradient. Using a combination of *in situ* and microarray hybridisation, Krol and colleagues identified cyclic gene networks of 40-100 genes for three distantly related vertebrate species: mouse (*Mus musculus*), chicken (*Gallus gallus*) and zebrafish (*Danio rerio*). Surprisingly, although the roles of Notch, fibroblast growth factor (FGF) and Wnt pathways were conserved, the only cyclic genes shared between all three species were orthologs of the Notch targets *Hes1* and *Hes5*. As above, this example underlines how the organisational level and scale at which a conserved phenotype is defined is important for identifying DSD. What is homologous across the compared species includes the vertebral column, somites and the somitogenesis process, but not identities of the segmentation clock genes (Oates et al., 2012; DiFrisco and Jaeger, 2021).

There are also known examples of DSD between human and mouse (summarised by Lynch, 2009). For example, Odom and colleagues compared four key transcription factors with conserved functions in the liver in human and mouse (FOXA2, HNF1A, HNF4A and HNF6), finding that 41-89% of the promoters bound by a given TF in one species were not bound by the same TF in the other. The divergence of binding sites included a site for HNF1A that is implicated in human diabetes and that is absent from the corresponding mouse region (Odom et al., 2007).

A study by Liao and Zhang could also be interpreted as an example of DSD between mouse and human. Comparing orthologous genes between the two species, they found that over 20% of human-essential genes (i.e. loss associated with infertility or death before puberty) were non-essential in mouse gene knockout experiments. Further analysis of gene paralogs in each species found that these essentiality differences were not caused by gene duplication, and were instead indicative of changes in the importance or roles of these genes in some conserved traits across mouse and human. The essentiality changes of these genes may have been partly driven by DSD (Liao and Zhang, 2008).

Another notable example of DSD in vertebrates is in sex determination. Cauret and colleagues report that there are at least seven distinct sex determination systems in pipid frogs, including evolutionary shifts between male and female heterogamy. In *X. laevis*, female differentiation is triggered by the gene *dm-w*, whereas it is present in males in other species, and was not detected at all in other species in the clade. For whatever reason, DSD appears to be especially frequent in sex-determination mechanisms (Haag and Doty, 2005; Cauret et al., 2019).

### Nematodes

One of the first documented cases of DSD was in nematode vulva development (see Haag and True, 2021). Although the phenotypic outcome of vulva development is crucial for nematode development and highly conserved across all nematodes, there is considerable and well-documented cryptic variation in the underlying genetic architecture. True and Haag (2001) identify *lin-39* mutant experiments performed by Eizinger and Sommer (1997) in *C. elegans* and *P. pacificus* as one of the earliest examples of DSD in vulva development. *lin-39* knockout mutants are vulvaless in both species, but with different cell fate outcomes: *C. elegans* mutants adopt a fusion cell fate, whereas in *P. pacificus* these cells instead undergo apoptosis.

Experiments in even more closely related species, such as those of Félix (2007) in *C. elegans*, *C. briggsae* and *C. remanei*, add further evidence of cryptic variation, with the same perturbations in cell ablation and gene overexpression experiments leading to different changes to cell fate outcomes. Kiontke and colleagues analysed over 40 characteristics of nematode vulva development across 51 different species, constructing a phylogeny that demonstrated high levels of variation and evolutionary changes across nematode evolution. (Kiontke et al., 2007). In addition, this evolutionary pattern was identified as biased for all but two characteristics, suggesting it may be adaptive DSD (see ‘Neutral versus adaptive DSD’).

This system has also been examined using computational approaches. Hoyos and colleagues constructed an ordinary differential equation-based computational model using known EGF and Notch pathway interactions in vulval development for six identical vulval precursor cells (Hoyos et al., 2011). By varying model parameters to reproduce different mechanisms, they were able to reproduce patterns of development for four different experimentally studied nematode species, highlighting that both experimental and computational approaches can be used to provide mechanistic insight into DSD occurrence.

The ease of RNAi screening in nematodes makes them highly amenable to large-scale gene knockdown approaches. Verster and colleagues constructed a *C. briggsae* RNAi library focusing on orthologues to *C. elegans* genes with a known RNAi phenotype (Verster et al., 2014). They then performed a large-scale RNAi knockdown screen of these 1333 orthologous genes in both species, identifying 679 genes that differed in loss-of-function phenotype between the two species, which was narrowed to 91 genes with a strong likelihood of differences in *in vivo* function. These 91 genes had undergone different patterns of DSD. Several differed in expression patterns due to promoter evolution, and transcription factors and recently evolved genes were more represented than core cell components. There was also a bias towards gene families with greater functional redundancy. As new RNAi protocols are developed for non-model nematode species, and other metazoa more broadly, it may be possible to further elucidate general patterns of DSD and incorporate it into our understanding of GRN evolution.

### Spiralia

In an example echoing that of *Drosophila* DV axis patterning, the annelid *Capitella teleta* has supplanted the ancestral role of BMP signalling in the establishment of the DV axis with another member of the transforming growth factor β (TGFβ) superfamily: the Activin/Nodal signalling pathway (Lanza and Seaver, 2020). The authors use antisense morpholino oligonucleotides to knock down transcription factors specific to both the BMP and Activin/Nodal signalling pathways, finding that knockdown of Activin/Nodal, but not BMP, led to loss of DV axis patterning. This contrasts with other spiralians, which primarily use BMP signalling to establish the DV axis. The authors suggest that, given the highly stereotypic nature of spiral cleavage across spiralians, this is an example of DSD.

### Tunicates

Lowe and Stolfi analysed gene expression patterns in the nervous system of the tunicate *Molgula occidentalis*, comparing them with the model tunicate *Ciona intestinalis*, which has a fully mapped larval connectome (Lowe and Stolfi, 2018). Both tunicate developmental cell lineages are highly stereotyped, with few apparent embryonic differences across deep evolutionary timescales. Lowe and Stolfi found that, although the spatial transcriptomic patterns of descending decussating neuron (ddN) development were largely conserved, the two species differed in the temporal dynamics of neuron transcription, with a key transcription factor required for ddN specification (Dmbx) expressed earlier in development relative to the mitotic exit of the neuron. In addition, they also tested whether the Dmbx driver *cis*-regulatory sequence of each species was able to drive ddN expression in the other. This was true for the *Ciona cis*-regulatory sequence in *Molgula*, but did not hold true when the *Molgula cis*-regulatory sequence was transplanted into *Ciona*, despite the overall homology of the sequences and their ability to drive identical gene expression patterns in each species. The authors attribute this asymmetry in *cis*-regulation to DSD.

## Detecting and modelling DSD

Although there is no standard way to detect DSD, with different studies often taking very different approaches, we suggest that these approaches can be grouped into three main categories: observational approaches that rely on measurements such as RNA-seq, perturbation approaches that manipulate the system and modelling approaches that use computational methods to describe how DSD can occur in a system. Most studies are not limited to one of these categories. The most robust methods generally rely on a combination of observational approaches and perturbation or modelling approaches to provide mechanistic insight into how DSD has occurred.

### Observational approaches

With the advent of single-cell RNA-sequencing (scRNA-seq) and dropping costs of long-read genome sequencing techniques, it has become feasible to study profiles of gene expression in non-model organisms without the gene manipulation techniques that can be difficult to implement in less well-studied systems. However, to date, this approach remains largely under-utilised outside model organisms (Miller et al., 2018; Alfieri et al., 2022). We now have the opportunity to fully use the potential of scRNA-seq to perform comparative analysis of genetic diversity across species. Using time-resolved data, developmental stages can be aligned between species based on gene expression and morphological changes, allowing measurement of any significant differences in what genes are expressed, when they are expressed or their levels and variability of expression.

Most recent experimental DSD studies we have described, such as Van Belleghem et al. (2023), Lanza and Seaver (2020) or Lowe and Stolfi (2018), make use of RNA-seq. This can be combined with other observational approaches, such as assay for transposase-accessible chromatin with sequencing (ATAC-seq) profiling to construct GRNs for the process being studied by identifying open chromatin regions (Van Belleghem et al., 2023) or *in situ* hybridisation to measure mRNA localisation and identify any differences in gene expression location (Lowe and Stolfi, 2018).

A limitation of observational approaches to detecting DSD is that they do not, on their own, link divergence of genes or gene expression with a conserved phenotype. Accordingly, we suggest the most successful approaches combine these kinds of observational techniques that identify potential DSD candidates with ‘perturbation’ or ‘modelling’ techniques that can suggest mechanisms for the occurrence of DSD. In addition, a complementary approach can be taken using methods from evolutionary genetics, which are often under-utilised in the evo-devo field. These are explored further below.

### Perturbation approaches

Early examples of DSD were mostly identified through ‘perturbation’ approaches. These include the cell ablation experiments used to identify DSD in nematode vulva development (Félix, 2007), which could be coupled with gene overexpression studies using vectors such as plasmids. The development of efficient RNAi knockdown and genetic modification techniques such as CRISPR/Cas editing systems has meant that modern approaches now include gene knockout and/or knockdown (Wotton et al., 2015; Verster et al., 2014; Lanza and Seaver, 2020), as well as reporter assays to assess the differential expression of genes or CREs in different species (Lowe and Stolfi, 2018). In addition, the availability of gene knockout databases has allowed knockout phenotypes to be more easily compared in a systematic way between species (Liao and Zhang, 2008).

To some extent, perturbation approaches can be considered a gold standard for the identification of DSD. When knocking out the same gene gives rise to different changes in phenotype in different species, this gives a good indication that the function of the gene in the organism has been altered over evolutionary time. Since the gene is linked to the change in phenotype, such experiments provide mechanistic insight into how DSD has occurred and which genes are involved. However, there is also a key disadvantage to relying on the perturbation approach. Many genetic knockdown tools are difficult to use outside the model organisms they have been developed for and tested in. Non-model organisms generally lack easily available genetic toolboxes, and while there are workarounds in some cases (such as the modification of *C. briggsae* to be responsive to RNAi feeding in Verster et al., 2014), perturbation approaches have more-limited application to detecting DSD in non-model organisms.

An easier method than attempting direct knockout or knockdown in a non-model organism may be to take a ‘gene transplant’ approach via the use of cross-species transgenic assays. In Lowe and Stolfi (2018), the authors used reporter plasmids to measure the cross-species compatibility of *cis*-regulatory sequences, finding that while a *Ciona cis*-regulatory sequence was able to activate gene expression in *Molgula*, the reverse was not true, despite highly similar patterns of gene expression. By demonstrating that differences in *trans*-regulation of specific genes had occurred, the authors were able to demonstrate DSD between *Ciona* and *Molgula* neuronal development in a manner that gave mechanistic insight rather than simply identifying that gene expression had changed with no underlying cause. These kinds of experimental approaches can be further complemented with a modelling approach.

### Modelling approaches

An alternative way to gain mechanistic insight into DSD involves modelling: using computational methods to complement observations, rather than perturbation approaches that may be difficult to apply outside model organisms. These can range from modelling theoretical gene networks involving only a few genes (Johnson and Porter, 2007; Baier et al., 2023) to approaches that combine network analysis with existing GRN data (Hoyos et al., 2011; Crombach and Jaeger, 2021). Modelling is ultimately required to integrate information about individual gene effects into complex network behaviours. Models validated by data thus provide the greatest degree of mechanistic insight into DSD occurrence.

One possibility for studying DSD is to use entirely mathematical or *in silico* modelling approaches. Schiffman and Ralph (2022) used a GRN model to simulate the dynamics of a set of interacting transcription factors, demonstrating that regulatory networks can undergo large amounts of change without disrupting the final phenotype or ‘fitness function’. Their theoretical model is largely based on a set of linear differential equations without the use of inputs from experimental data. An approach based on genotype-phenotype mapping is taken by Khatri and Goldstein (2019), who developed a model that accounts for mutation, selection and genetic drift to explain how DSD can occur in spatial patterning. Finally, Crombach and Jaeger (2021) proposed the use of *in silico* evolution to study DSD in the insect gap gene network, a simulation-based approach that models the genotype changes of digital organisms over many generations, which can then be translated into a phenotype. The increasing availability of computational platforms to study *in silico* evolution such as Aevol, which allows evolutionary simulations of digital organisms under different conditions (Liard et al., 2020), is making these methods more feasible.

The difficulty, of course, lies in ensuring that the models reflect biological reality. A model developed without reference to previously studied biological systems can easily be too simplistic, generic or idealised to capture relevant system behaviours. Fortunately, we are increasingly seeing modelling software that can take advantage of the newfound abundance of scRNA-seq data. An example is CellOracle, which uses scRNA-seq and ATAC-seq data to construct putative GRNs that can be perturbed *in silico* (Kamimoto et al., 2023). Such tools will better enable integration of modelling with observational approaches in the future.

### Evolutionary methods

Since DSD is an evolutionary process, any attempts to detect or quantify it must take the evolutionary history of the trait and species being examined into account. While most studies of DSD will consider the relevant trait phylogeny, few studies make full use of approaches from evolutionary genetics and comparative phylogenetic research, sticking instead to common developmental biology methods such as scRNA-seq analysis. This is part of a wider tendency noted in the evo-devo literature (Parsons and Albertson, 2013; Artieri and Singh, 2010).

A key evolutionary consideration is that DSD can be driven by selection (see section ‘Neutral versus adaptive DSD’). As a result, the detection of selection in genomic regions correlated with conserved traits may be indicative of DSD through compensatory adaptive evolution. One way in which selection can be detected in coding regions is through variations on the McDonald-Kreitman test, using tools such as asymptoticMK (Haller and Messer, 2017; McDonald and Kreitman, 1991). The McDonald-Kreitman test differentiates between directional selection, stabilising selection and neutral evolution, based on the ratio between non-synonymous and synonymous substitutions in coding regions. Selection can also be detected through the use of ‘selection signature QTLs’ or ssQTLs, which combine QTL mapping and population genomics to identify loci associated with a specific phenotype that is under selection. This enables comparison of trait-associated loci across species (Parsons and Albertson, 2013). Despite its inability to detect neutral DSD, ssQTL mapping can be a valuable complement to the observational approaches discussed above.

## Discussion

In the past few decades, experimental progress in probing the genetic basis of development has led to the unexpected finding that cryptic developmental diversity underlies many conserved characters. DSD has the consequence that genetic conservation cannot be straightforwardly assumed from phenotypic homology. This poses a challenge for extrapolating findings from model organisms in general, as well as for translation to human biology in particular (Lynch, 2009). At the same time, it is also an opportunity to study the evolutionary lability of developmental gene regulation.

In our view, the key to accommodating DSD within comparative developmental biology is through deeper integration with evolutionary biology. Specifically, if we can arrive at an understanding of DSD as a regular evolutionary process, as opposed to a disconnected series of contingent events, this understanding can inform the formulation of null hypotheses for how much genetic divergence to expect on the basis of phylogenetic distance (Church and Extavour, 2020; DiFrisco, 2023). It is not unrealistic to expect that the evo-devo field can arrive at this state with a combination of better theory and better use of data.

From the theory side, a central requirement is an improved understanding of the guiding principles of GRN evolution (Peter and Davidson, 2011; Rebeiz et al., 2015; Halfon, 2017). It is likely that DSD often occurs in developmental GRNs that are robust to genetic change. The robustness of a GRN is a dynamical property that depends not just on network topology but also on gene expression rates, timings, interaction strength and boundary conditions (Jiménez et al., 2017a,b; Verd et al., 2019; DiFrisco and Jaeger, 2019). Together, these factors determine the scope and type of genetic changes that a GRN can tolerate without disrupting its phenotypic output. Improved understanding of DSD can come from the further characterisation of how different classes of dynamical GRNs confer robustness to genetic change in some parts of a network and sensitivity in other parts (Cotterell and Sharpe, 2010; Steinacher et al., 2016; Jiménez et al., 2017a,b).

These theoretical modelling approaches have limitations, however. They have often been restricted to three-gene networks for mathematical tractability, and their empirical validation requires a level of biological knowledge and data quality that is currently available for only a small number of well-studied systems, such as the gap gene segmentation cascade (Crombach and Jaeger, 2021). A further difficulty is that well-studied developmental GRN architectures tend to have strong epistatic effects and gene numbers that are intermediate between the large number of loci assumed by the infinitesimal model (Fisher, 1918; Barton et al., 2017) and few-loci models (Crow and Kimura, 2009), which presents a general challenge for incorporating GRNs into quantitative evolutionary genetics.

From the data side, the widespread collection of RNA-seq data provides an unprecedented opportunity to map the phylogenetic diversity of gene expression. Computational methods for inferring GRNs from transcriptomic data and other kinds of data, such as ATAC-seq, are proliferating (Badia-i-Mompel et al., 2023; Kamimoto et al., 2023; Van den Broeck et al., 2020). However, a key obstacle continues to be the lack of systematic identification of *cis*-regulatory elements (Halfon, 2017). This will be crucial for informing our understanding of the principles of GRN evolution. Even when paired with more reliable GRN inference algorithms, however, RNA-seq data alone is not sufficient for detecting DSD. RNA-seq data does not causally link divergent transcripts with any conserved phenotype. Hypothetical inferred GRNs still must be validated experimentally, which remains difficult in non-model organisms. As a result, computational approaches based on GRN inference and *in silico* perturbation may soon be able to suggest many more cases of potential DSD than can be efficiently validated.

Integration with evolutionary biology goes in both directions. DSD is not only a product of evolutionary processes, but also has evolutionary consequences. DSD between reproductively isolated populations likely contributes to genetic incompatibilities that cause hybrid non-viability or infertility, also known as Bateson-Dobzhansky-Muller incompatibility (Bateson, 1909; Dobzhansky, 1936; Muller, 1942; Johnson and Porter, 2000; Khatri and Goldstein, 2019; Schiffman and Ralph, 2022). Simulations and theoretical modelling have suggested that speciation due to DSD will be more rapid when directional selection is involved (Porter and Johnson, 2002; Tulchinsky et al., 2014) but it may also happen under strictly neutral evolution (Schiffman and Ralph, 2022).

Another evolutionary consequence of DSD relates back to robustness and evolvability (see Glossary, Box 1), defined as the capacity to generate potentially adaptive variations (Hansen et al., 2023). Phenotypic robustness allows genetic systems to explore spaces of variation without the constraint that each stepwise change must confer an adaptive benefit (Wagner, 2005; 2008). When a phenotype is highly sensitive to genetic change, it may confine the population to local fitness optima. In contrast, phenotypic robustness can allow populations to spread across large ‘neutral networks’ of genetic variation (Gavrilets, 2004). Accumulated cryptic variation can eventually be released, due to environmental change or novel mutations, to drive phenotypic change (Payne and Wagner, 2019; Schmalhausen, 1949), which may enable populations to access more global fitness optima. This is the general rationale for why phenotypic robustness is thought to enhance evolvability (Wagner, 2005; Manrubia et al., 2021). When the above process of robustness-enhanced evolution occurs, DSD corresponds to the intermediate step of accumulation of cryptic variation.

An interesting question for further study is whether, similar to robustness, stabilising selection on a developmental-genetic system with many underlying degrees of freedom has the same consequences for evolvability. Both can be mechanisms of DSD. Deeper understanding of these mechanisms will go hand-in-hand with improved methods of detection and wider sampling of taxonomic diversity in the project of determining the relative probability and actual frequency of DSD.
